# Stereotactic body radiotherapy for bone-only oligometastatic bladder Cancer: a multi-institutional experience

**DOI:** 10.1016/j.ctro.2026.101246

**Published:** 2026-07-30

**Authors:** Burak Tilki, Ozan Cem Guler, Birhan Demirhan, Pervin Hurmuz, Ayşenur Elmalı, Tolunay Kumru, Gokhan Ozyigit, Melek Yavuz, Cem Onal

**Affiliations:** a Department of Radiation Oncology, University of Health Sciences, Antalya City Hospital, Antalya, Turkiye; b Department of Radiation Oncology, Baskent University Faculty of Medicine, Adana Dr. Turgut Noyan Research and Treatment Center, Adana, Turkiye.; c Division of Radiation Oncology, Iskenderun Gelisim Hospital, Hatay, Turkiye.; d Department of Radiation Oncology, Hacettepe University Faculty of Medicine, Ankara, Turkiye.; e Department of Radiation Oncology, Baskent University Faculty of Medicine, Ankara, Turkiye.

**Keywords:** Bladder cancer, Bone metastasis, Oligometastatic disease, Stereotactic body radiotherapy, Metastasis-directed therapy

## Abstract

**Purpose:**

To evaluate clinical outcomes, patterns of failure, local control (LC), and time to next systemic therapy (TTNT) in patients with bone-only oligometastatic bladder cancer treated with stereotactic body radiotherapy (SBRT).

**Materials and Methods:**

We retrospectively analyzed 26 patients with 41 bone metastases treated with SBRT between 2012 and 2020 across four institutions. All patients had ≤3 bone metastases and received metastasis-directed therapy to all lesions. Overall survival (OS), progression-free survival (PFS), TTNT, and LC were estimated using the Kaplan–Meier method.

**Results:**

After a median follow-up of 97.8 months, median OS was 8.9 months, with 1- and 2-year OS rates of 38.5% and 22.4%, respectively. Median PFS was 5.8 months, with 1- and 2-year PFS rates of 29.4% and 25.2%, respectively. Median TTNT was 4.4 months, and only 19.8% of patients remained free from systemic therapy at 1 year. Disease progression occurred in 46.2% of patients and was predominantly distant metastasis. In contrast, LC was excellent, with 1- and 2-year rates of 94.7% and 88.0%, respectively. No grade ≥ 3 toxicities were observed.

**Conclusion:**

SBRT achieved excellent LC with minimal toxicity. However, systemic progression remained common. These findings suggest that prospective studies are needed to determine the role of SBRT within multimodal treatment strategies for bone-only oligometastatic bladder cancer.

## Highlights

• SBRT achieved excellent local control in bone-only oligometastatic BCa.
• Distant metastasis was the dominant pattern of failure after SBRT.
• Median progression-free survival was only 5.8 months.
• Time to next systemic therapy remained short despite MDT.
• Bone-only oligometastatic BCa may represent systemic disease.

## 1 Introduction

Bladder cancer (BCa) is among the most common malignancies of the urinary tract and is associated with poor outcomes in the metastatic setting [1]. Nearly half of patients treated with curative intent ultimately develop disease progression, highlighting the aggressive nature of this disease [2], [3], [4]. Despite advances in systemic therapies, including immune checkpoint inhibitors and antibody–drug conjugates, long-term survival remains limited [5], [6], [7].

The concept of oligometastatic disease, first proposed by Hellman and Weichselbaum, describes an intermediate clinical state between localized and widely metastatic cancer, in which a limited number of metastatic lesions may be amenable to curative-intent local therapy [8]. In this context, metastasis-directed therapy (MDT), including surgery and stereotactic body radiotherapy (SBRT), has emerged as a promising strategy to improve disease control and potentially delay systemic progression. SBRT enables the delivery of highly conformal, high-dose radiation in a limited number of fractions and has demonstrated excellent local control (LC) across multiple tumor types.

However, the biological and clinical relevance of the oligometastatic paradigm in BCa remains uncertain. Unlike prostate or renal cell carcinoma, where oligometastatic disease may reflect a more indolent phenotype, BCa is generally characterized by aggressive systemic behavior [2], [6]. Existing studies evaluating SBRT in oligometastatic BCa are limited, frequently include heterogeneous patient populations with mixed metastatic sites, and rarely focus on site-specific patterns of disease dissemination [2], [4], [9], [10], [11].

Bone metastases occur in approximately 30–40% of patients with advanced BCa and represent the second most common site of dissemination after lymph nodes [11], [12]. However, patients presenting with bone-only oligometastatic disease constitute a distinct and poorly characterized subgroup. It remains unclear whether this presentation reflects a biologically favorable oligometastatic state or an early manifestation of systemic dissemination. Although SBRT has demonstrated high rates of LC in oligometastatic urothelial cancer, its ability to alter the systemic disease course remains uncertain. In particular, whether MDT can meaningfully delay the need for systemic therapy is of major clinical relevance, yet this endpoint has been insufficiently explored in the literature.

Therefore, in this multi-institutional study, we evaluated clinical outcomes, including overall survival (OS), progression-free survival (PFS), time to next systemic therapy (TTNT), and LC, in patients with bone-only oligometastatic BCa treated with SBRT. We further assessed patterns of failure and the durability of disease control following metastasis-directed therapy.

## 2 Materials and methods

### 2.1 Study design and patient selection

This multi-institutional retrospective study included patients with histologically confirmed BCa treated with SBRT for bone-only oligometastatic disease between January 2012 and December 2020 across four tertiary centers.

Eligibility criteria were: (i) histopathological confirmation of BCa; (ii) presence of ≤3 bone metastases without evidence of visceral or nodal metastatic disease at the time of SBRT; (iii) prior definitive treatment of the primary tumor; and (iv) treatment of all identified metastatic lesions with SBRT using fraction doses ≥5 Gy in accordance with AAPM Task Group 101 recommendations [13]. Patients with induced oligometastatic disease were eligible only if all active metastatic lesions were confined to bone at the time of SBRT. Patients who underwent surgical resection of metastatic lesions, had additional non-bone metastatic disease, or did not receive definitive treatment for the primary tumor were excluded. At the time of SBRT, all patients had controlled primary bladder tumors following definitive local treatment, with no evidence of persistent or recurrent intravesical disease.

The oligometastatic disease state was further classified according to the ESTRO/EORTC consensus framework as de novo oligometastatic, oligorecurrent, or induced oligometastatic disease. The timing of systemic therapy prior to SBRT was recorded and categorized as neoadjuvant, adjuvant, or treatment for metastatic progression before SBRT.

Clinical, imaging, treatment, and follow-up data were collected from institutional databases. The study was conducted in accordance with the Declaration of Helsinki and approved by the local institutional review boards.

### 2.2 Radiotherapy planning and delivery

Non-contrast computed tomography (CT) simulation was performed in the supine position using appropriate immobilization devices (BodyFIX®, Elekta), with CT images acquired at a slice thickness of 1–2 mm. Target volumes were delineated on planning CT with fusion of available diagnostic imaging, including magnetic resonance imaging (MRI) or fluorodeoxyglucose positron emission tomography (FDG-PET/CT), when indicated.

The gross tumor volume (GTV) was defined as the visible metastatic lesion. A planning target volume (PTV) margin was applied according to institutional protocols to account for setup uncertainties and organ motion.

Dose and fractionation schedules were individualized based on lesion size, anatomical location, and proximity to organs at risk (OARs). SBRT was delivered in 1–5 fractions using image-guided radiotherapy techniques. The prescribed dose was typically normalized to ensure coverage of at least 95% of the PTV while prioritizing adherence to OAR dose constraints.

Treatment plans were optimized to achieve adequate target coverage while respecting predefined organ-at-risk dose constraints. The planning objectives and dose constraints are summarized in Supplementary Table 1.

The biologically effective dose (BED10) was calculated using the linear–quadratic model:

$${\mathrm{BED}}_{10}=\mathrm{nd}\;\left(1+\frac{d}{10}\right)$$

where *n* is the number of fractions and d is the dose per fraction. BED10 was calculated separately for each treated lesion and summarized at the lesion level.

Daily image guidance with cone-beam CT (CBCT) was used for patient positioning and verification, with six-degree-of-freedom corrections applied before each fraction.

### 2.3 Follow-up and outcome assessment

Patients were followed at regular intervals with clinical evaluation and imaging according to institutional protocols. Imaging modalities included CT, MRI, or FDG-PET/CT, as clinically indicated. Patterns of failure were categorized as local, distant, or combined progression.

LC was defined as the absence of progression within the irradiated lesion. Local failure was defined as radiological progression within the treated lesion based on serial imaging, including CT, MRI, and FDG-PET/CT when available. Because many treated bone lesions were non-measurable according to RECIST 1.1, assessment was based on multidisciplinary radiological interpretation rather than RECIST criteria alone. PFS was defined as the time from completion of SBRT to the first occurrence of local progression, distant metastasis (DM), or death from any cause. OS was calculated from completion of SBRT to death or last follow-up. For patients with metachronous disease, the metastasis-free interval was defined as the time from completion of definitive primary treatment to radiological diagnosis of metastatic disease. The interval from oligometastatic diagnosis to MDT was defined as the time from radiological diagnosis of oligometastatic disease to initiation of SBRT and was calculated for all patients. Time to next systemic therapy (TTNT) was defined as the interval from completion of SBRT to initiation of a new systemic treatment line or escalation of an existing systemic regimen, irrespective of whether documented radiological or clinical progression preceded the treatment change. Continuation of an unchanged systemic regimen during or after SBRT was not considered an event. Patients who did not initiate or escalate systemic therapy were censored at the date of last follow-up or death.

Treatment-related adverse events were graded according to the Common Terminology Criteria for Adverse Events (CTCAE), version 5.0.

### 2.4 Statistical analysis

Statistical analyses were performed using SPSS version 25.0 (IBM Corp., Armonk, NY, USA) and GraphPad Prism version 9.3.1 (GraphPad Software, San Diego, CA, USA). Descriptive statistics were used to summarize patient, tumor, and treatment characteristics. OS, PFS, TTNT, and LC were estimated using the Kaplan–Meier method. Comparisons between groups were performed using the log-rank test. Univariate analyses were conducted to evaluate the association between clinical and treatment-related variables, including age (<65 vs ≥65 years), number of metastases (1 vs >1), staging modality (FDG-PET/CT vs conventional imaging), interval from oligometastatic diagnosis to MDT (<6 vs ≥6 months), prior systemic therapy before SBRT, and SBRT dose (BED10 < 50 Gy vs ≥50 Gy). Given the exploratory nature of the study and the limited sample size, multivariable analyses were not performed. A two-sided *p*-value <0.05 was considered statistically significant.

## 3 Results

### 3.1 Patient characteristics

Baseline clinical and tumor characteristics are summarized in Table 1. A total of 26 patients with 41 bone metastases treated with SBRT were included in the analysis. The median age was 66 years (range, 39–79), and the majority of patients were male (92.3%).

**Table 1:** Baseline patient and tumor characteristics.

Characteristics	Number	%
Age (years), (median, range)	66 (39–79)	
Gender		
Male	24	92.3
Female	2	7.7
Oligometastatic disease state		
De novo oligometastatic	13	50
Oligorecurrent	9	34.6
Induced oligometastatic	4	15.4
Number of metastases		
1	15	57.7
2	7	26.9
3	4	15.4
Staging		
FDG-PET/CT	18	69.2
Conventional imaging	8	30.8
Site of metastasis ⁎		
Spine	22	53.7
Non-spine	19	46.3
Treatment of primary tumor		
Surgery	17	65.4
Chemoradiotherapy	8	30.7
Surgery + Chemoradiotherapy	1	3.9
Any systemic therapy before SBRT		
Yes	14	53.8
No	12	46.2
Initiation or escalation of systemic therapy after SBRT		
Yes	16	61.5
No	10	38.5

Abbreviations: FDG-PET/CT: fluorodeoxyglucose positron emission tomography, MDT: metastasis-directed therapy.

⁎ Reported per treated lesion.

At staging, 69.2% of patients underwent FDG-PET/CT, while the remaining patients were evaluated with conventional imaging. Metastatic presentation was evenly distributed, with 13 patients (50%) presenting with synchronous disease and 13 (50%) with metachronous disease. According to the ESTRO/EORTC classification, these corresponded to 13 de novo, 9 oligorecurrent, and 4 induced oligometastatic cases. Among patients with metachronous disease, the median metastasis-free interval was 6.6 months (IQR, 1.8–18.2 months). Most patients had a limited metastatic burden, with a single bone metastasis observed in 57.7% of cases, while 26.9% and 15.4% had two and three lesions, respectively. The spine was the most common metastatic site (22 lesions, 53.7%), followed by the pelvis (14 lesions, 34.1%), ribs (3 lesions, 7.3%), and sternum (2 lesions, 4.9%).

Before SBRT, 14 patients (53.8%) had received systemic therapy, including 3 patients (11.5%) who received neoadjuvant therapy and 11 patients (42.3%) who received adjuvant therapy following definitive treatment of the primary tumor. No patient received systemic therapy for metastatic progression before SBRT.

A total of 18 lesions (44%) were treated with single fraction, while 23 lesions (56%) were treated with multiple fractions due to the unfavorable location and larger tumor size. Total radiation doses and fraction numbers are summarized in Table 2.

**Table 2:** Total radiation doses in Gray (Gy) and fraction numbers.

Total dose/fraction number	Number	%
16 Gy/1	7	17.2
18 Gy/1	11	26.8
17 Gy/2	1	2.4
20 Gy/2	6	14.6
24 Gy/2	2	4.9
25 Gy/5	3	7.3
27 Gy/3	1	2.4
30 Gy/5	6	14.6
35 Gy/5	4	9.8

### 3.2 Treatment outcomes

**Fig. 1:**
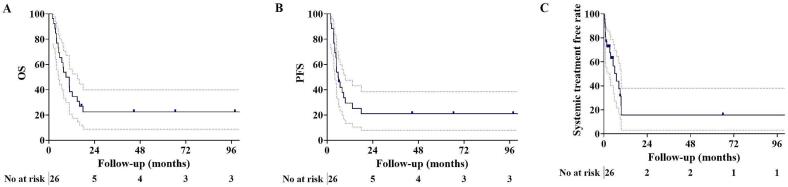
Kaplan–Meier curves for (A) overall survival (OS), (B) progression-free survival (PFS), and (C) time to next systemic therapy (TTNT).

Median follow-up was estimated using the reverse Kaplan–Meier method and was 97.8 months (95% CI, 38.5–157.1). However, only three patients remained under observation beyond 72 months. The median OS was 8.9 months (95% CI, 4.8–13.0), with 1- and 2-year OS rates of 38.5% and 22.4%, respectively (Fig. 1A). Similarly, median PFS was 5.8 months (95% CI, 3.4–8.2), with corresponding 1- and 2-year PFS rates of 29.4% and 25.2%, respectively (Fig. 1B). All deaths occurred after documented disease progression; therefore, no PFS events were attributable to death without prior progression.

Disease progression occurred in 12 patients (46.2%) at a median of 5.4 months (95% CI, 3.8–8.5) following completion of MDT. Among the 12 patients who experienced disease progression, two developed oligometastatic progression, whereas 10 developed polymetastatic progression. Repeat SBRT was not administered to patients who developed subsequent oligometastatic progression. Further treatment was individualized based on multidisciplinary evaluation.

All patients who experienced progression developed DM. Among these, only two patients also experienced concurrent in-field recurrence, whereas isolated local failure was not observed. In contrast, LC remained excellent, with 1- and 2-year LC rates of 94.7% and 88.0%, respectively. Only two local failures were observed among the 41 treated lesions, both occurring concurrently with distant disease progression. Although the estimated 2-year local-control rate was 88.0%, this estimate should be interpreted cautiously given the limited number of local events and the substantial competing mortality.

### 3.3 Time to next systemic therapy

During follow-up, 16 patients (61.5%) initiated a new systemic treatment or underwent escalation of their existing treatment after SBRT. Of these, 12 patients initiated or escalated systemic therapy following documented radiological or clinical progression, whereas 4 patients received treatment without documented progression. The median TTNT was 4.4 months (95% CI, 1.5–8.4), and the 1-year systemic treatment-free rate was 19.8% (Fig. 1C).

**Fig. 2:**
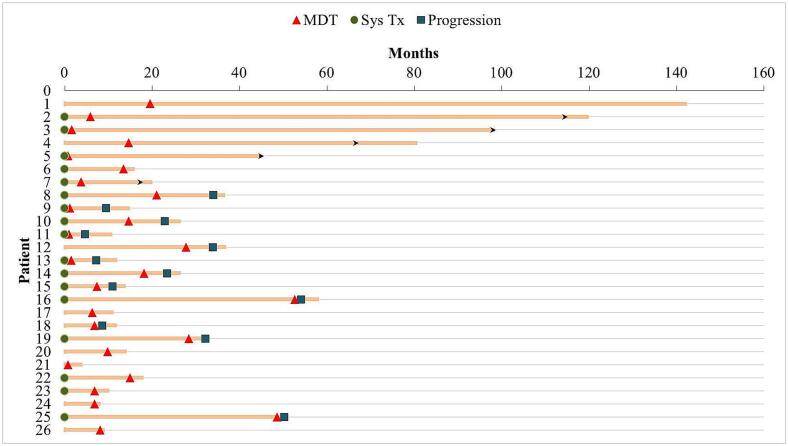
Swimmer plot illustrating the clinical course of individual patients. Time 0 represents the diagnosis of metastatic disease. Each bar depicts the interval from metastatic diagnosis to last follow-up or death, with the timing of SBRT, systemic therapies, disease progression, and survival status indicated.

Among patients requiring systemic therapy, the majority received platinum-based chemotherapy (*n* = 11), while a smaller proportion received carboplatin–paclitaxel (*n* = 3), vinflunine (n = 1), or immune checkpoint inhibitors (pembrolizumab or atezolizumab; n = 1 each) (Fig. 2).

### 3.4 Prognostic factors

Univariate analyses did not identify any statistically significant predictors of OS or PFS (Table 3). Although several variables showed directional associations with outcome, none reached statistical significance, likely due to the limited sample size and number of events.

**Table 3:** Univariate analysis for overall survival and progression-free survival.

Characteristics	*n*	OS HR (95% CI)	*p*	PFS HR (95% CI)	*p*
Age					
< 65 y	12	1	0.39	1	0.32
≥ 65 y	14	1.48 (0.60–3.63)		1.59 (0.65–3.90)	
Number of metastases					
1	15	1	0.56	1	0.74
>1	11	1.30 (0.54–3.18)		1.16 (0.47–2.88)	
Interval from oligometastatic diagnosis to MDT					
< 6 mo	13	1	0.23	1	0.37
≥ 6 mo	13	1.72 (0.71–4.16)		1.50 (0.62–3.63)	
Staging					
FDG-PET/CT	18	1	0.79	1	0.14
Conventional imaging	8	1.13 (0.45–2.84)		2.37 (0.76–7.36)	
Prior systemic therapy before SBRT					
Yes	14	1	0.15	1	0.34
No	12	1.92 (0.79–4.66)		1.55 (0.63–3.81)	
Time between oligometastasis and MDT					
< 2 mo	12	1	0.29	1	0.23
≥ 2 mo	14	1.63 (0.66–4.02)		1.75 (0.71–4.33)	
BED10					
< 50 Gy	12	1	0.21	1	0.29
≥ 50 Gy	14	1.78 (0.72–4.38)		1.83 (0.74–4.51)	

Abbreviations: BED10: biologically effective dose 10 Gy, CI = confidence interval, HR = hazard ratio, Gy: gray, MDT: metastasis-directed therapy, OS: overall survival, PFS: progression-free survival.

### 3.5 Toxicity

Treatment-related toxicity was generally mild. Grade 2 pain flare occurred in 5 patients (19.2%), while grade 2 esophagitis was observed in 4 patients (15.4%). All events resolved with conservative medical management. No grade 3 or higher acute treatment-related toxicities, treatment-related deaths, or clinically significant late toxicities were observed during follow-up. During follow-up, no vertebral compression fractures, pathological fractures, malignant spinal cord compression, or re-irradiation were identified.

## 4 Discussion

In this multi-institutional retrospective study, we evaluated outcomes of SBRT in a multicenter cohort of patients with bone-only oligometastatic BCa. While SBRT achieved high LC, survival outcomes remained poor, characterized by early disease progression and a short TTNT. Collectively, these findings suggest that bone-only oligometastatic BCa may represent an early manifestation of systemic disease rather than a biologically indolent oligometastatic state. Importantly, SBRT was well tolerated, with no observed grade ≥ 3 toxicities.

The role of MDT in oligometastatic BCa remains controversial. Surgical metastasectomy has been explored in carefully selected patients, with reported 5-year survival rates ranging from 28% to 60% [14], [15], [16]. However, these outcomes are likely influenced by substantial selection bias, as candidates for surgery typically represent a favorable subgroup. SBRT has emerged as a non-invasive local treatment option (Table 4) [3], [9], [10], [17], [18], [19], [20]. Prior studies have consistently demonstrated excellent local tumor control following SBRT [3], [10]. Our findings were comparable but should be interpreted with caution. Only two local failures were observed, both occurring concurrently with distant progression, while the limited survival of many patients reduced the opportunity for long-term assessment of LC. Furthermore, assessment of local progression in bone is inherently challenging, particularly in sclerotic lesions that are not reliably measurable according to RECIST 1.1. Accordingly, the reported LC rates should be considered exploratory rather than definitive evidence of durable local efficacy.

**Table 4:** Summary of published studies evaluating stereotactic body radiotherapy (SBRT) in metastatic bladder cancer.

Author	Year, design	*n*	Metastasis site	Median BED10 (Gy)	Median OS (mo)	Median PFS (mo)	LC (%)	Toxicity
Leonetti A et al. (17)	2018, R	7	Mixed	48	14.9	2.9	100 (1-year)	≥ Grd 2: none
Augugliaro M et al. (18)	2018, R	13	Mixed	35.7	–	4.2	57 (4-months)	≥ Grd 2: none
Francolini G et al. (9)	2019, R	19	Mixed	48	13.8	5.6	68 (1-year)	≥ Grd 3: none
Franzese C et al. (3)	2020, R	61	Mixed	78.7	25.6	10.1	88.9 (2-year)	≥ Grd 2: none
Miranda AF et al. (19)	2021, R	52	Mixed	–	51	8	72 (1-year)	≥ Grd 3: 4%
Spaas M et al. (20)	2023, P	32	Mixed	43.2	14.3	4.4	76 (1-year)	≥ Grd 3: 18%
Svedman FC et al. (10)	2024, R	39	Mixed	146	26.2	4.1	85 (2-year)	≥ Grd 3: none
Present study	2026, R	26	Bone-only	48	8.9	5.8	88 (2-year)	≥ Grd 3: none

Abbreviations: BED10: biologically effective dose 10 Gy, Grd: grade, LC: local control, n: patient number, OS: overall survival, PFS: progression-free survival, P: prospective, R: retrospective, RT: radiotherapy.

OS in our cohort was shorter than that reported in several contemporary SBRT series [3], [9], [10], [17], [18], [19], [20]. These differences should be interpreted with caution because of the retrospective design, small sample size, prolonged study period, and treatment heterogeneity. In addition, several important prognostic factors, including performance status, comorbidity burden, overall metastatic burden, and prior treatment history, were unavailable, limiting adjustment for residual confounding. Therefore, our findings should not be interpreted as evidence that bone-only oligometastatic urothelial carcinoma is inherently associated with a poorer prognosis. Furthermore, most patients were treated before the widespread adoption of contemporary first-line therapies, including enfortumab vedotin plus pembrolizumab, limiting direct comparison with modern metastatic urothelial carcinoma cohorts.

Baseline staging may also have influenced the observed outcomes. Nearly one-third of patients were staged using conventional imaging rather than FDG-PET/CT, potentially leaving occult metastatic disease undetected at the time of SBRT. Although staging modality was not significantly associated with PFS or OS in this exploratory analysis, patients staged with conventional imaging showed a directional trend toward inferior PFS. This finding should be interpreted cautiously given the limited sample size and wide confidence interval but raises the possibility that baseline understaging contributed to earlier disease progression in some patients.

Despite excellent local tumor control, systemic progression remained the predominant pattern of failure. In our cohort, all patients with disease progression developed distant metastases, and isolated in-field recurrence was not observed. This pattern is consistent with previous reports showing that most failures following SBRT for oligometastatic BCa occur outside the treated field [9], [10]. Twelve patients (46.2%) experienced progression after a median of 5.4 months, suggesting that some patients develop early systemic relapse despite effective local treatment. However, given the retrospective design, treatment heterogeneity, and potential residual confounding, it remains uncertain whether these outcomes reflect intrinsic tumor biology, patient selection, staging limitations, or other unmeasured prognostic factors. Accordingly, these observations should be regarded as hypothesis-generating and require prospective validation.

This pattern raises the possibility that bone-only oligometastatic BCa may not represent a true oligometastatic phenotype in all patients. It is also consistent with the recognized propensity of urothelial carcinoma for early systemic dissemination and occult micrometastatic disease [21], [22]. Bone-only metastasis may therefore represent an early phase of more widespread disease rather than a distinct curable state. These findings underscore the importance of integrating effective systemic therapy with carefully selected metastasis-directed treatment.

Another important consideration is the role of radiation dose. While prior studies suggest that higher BED may improve LC, inadequate dosing has been associated with inferior outcomes [18]. In the present study, the median lesion-level BED10 was 48.0 Gy, and excellent LC was observed despite substantial heterogeneity in dose and fractionation. However, no significant association between BED and survival outcomes was observed. This finding should be interpreted cautiously, given the limited sample size and potential lack of statistical power, and suggests that local dose escalation alone is unlikely to overcome the underlying systemic disease biology.

Dose and fractionation varied across participating institutions, reflecting individualized treatment planning based on lesion location, proximity to critical organs, prior irradiation, and institutional practice. Accordingly, not all treatment regimens fulfilled contemporary BED-based definitions of ablative SBRT. Moreover, BED-based comparisons should be interpreted cautiously, particularly for single-fraction SBRT, because the conventional linear-quadratic model has recognized limitations at very high doses per fraction and may not fully capture the biological effects of these regimens. Therefore, the LC outcomes should be interpreted in the context of this treatment heterogeneity.

The median TTNT of 4.4 months was shorter than the 12.1 months reported by Franzese et al. [3]. However, this comparison should be interpreted cautiously because the study populations and clinical contexts differed substantially. Our cohort included patients with de novo, oligorecurrent, and induced oligometastatic disease, and more than half had received systemic therapy before SBRT. Consequently, TTNT reflected different clinical settings. In treatment-naïve patients, it represented initiation of first-line systemic therapy, whereas in previously treated patients it reflected escalation to a subsequent treatment line rather than intentional deferral of systemic therapy. Therefore, TTNT should not be interpreted as a direct measure of the ability of SBRT to delay systemic therapy across all clinical settings.

These findings have several important clinical implications. First, although SBRT provides excellent local tumor control, it should not be considered a definitive treatment strategy when used in isolation. Rather, its primary role may be to consolidate limited sites of disease within a broader multimodality treatment approach. Second, the short TTNT observed in this study highlights the importance of early integration of systemic therapy. The therapeutic landscape of metastatic urothelial carcinoma has evolved substantially since most patients in the present cohort were treated. Immune checkpoint inhibitor-based combination therapy, particularly enfortumab vedotin plus pembrolizumab, has redefined first-line treatment, while antibody–drug conjugates and biomarker-directed therapies, including FGFR-targeted agents, have further expanded therapeutic options [5], [6], [23], [24]. Emerging HER2-directed therapies may further refine treatment strategies in selected patients. These advances are likely to influence both patient selection for metastasis-directed therapy and expected clinical outcomes. Accordingly, prospective studies evaluating SBRT alongside contemporary systemic therapies are needed to better define its role within multidisciplinary management. Finally, improved patient selection remains critical. Emerging biomarkers, such as circulating tumor DNA (ctDNA), genomic profiling, and advanced imaging approaches, including radiomics, may help identify patients with truly limited disease who are most likely to benefit from MDT.

Despite the relatively short median PFS, the swimmer plot demonstrated that a subset of patients achieved durable disease control lasting several years following SBRT, with some remaining free from additional systemic therapy for prolonged periods. This heterogeneity suggests that oligometastatic bladder cancer may encompass distinct biological phenotypes, including a subgroup with a more indolent disease course that may derive durable benefit from metastasis-directed therapy. However, our cohort was too small to identify clinical factors associated with these favorable outcomes. Future prospective studies integrating clinical, imaging, and molecular biomarkers are needed to identify patients most likely to achieve durable benefit from SBRT.

This study has several limitations. Its retrospective multicenter design, small sample size, and inherent selection bias limit the generalizability of the findings. The absence of a control group precludes determination of the independent impact of SBRT on survival. Heterogeneity in SBRT dose-fractionation schedules and systemic therapies may also have influenced outcomes, while conventional BED calculations should be interpreted cautiously for high-dose single-fraction SBRT. Furthermore, several important prognostic variables, including performance status, histological subtype, detailed prior systemic therapy, treatment response, and lesion volume, were unavailable, limiting adjustment for residual confounding. No vertebral compression fractures, pathological fractures, malignant spinal cord compression, or re-irradiation were identified during follow-up. However, these outcomes were not prospectively or systematically collected because of the retrospective multicenter design, and incomplete ascertainment cannot be excluded. Nevertheless, all patients had controlled primary bladder tumors at the time of SBRT following definitive local treatment, consistent with the eligibility criteria of several landmark randomized trials evaluating metastasis-directed therapy. This enhances the clinical relevance and comparability of our cohort within the contemporary oligometastatic treatment paradigm. Despite these limitations, the multi-institutional design and focus on the rare and underexplored subgroup of patients with bone-only oligometastatic bladder cancer represent important strengths of the present study.

## 5 Conclusion

SBRT for bone-only oligometastatic bladder cancer provided excellent LC with minimal toxicity but was associated with relatively short progression-free survival and time to next systemic treatment, highlighting the predominance of systemic disease progression. These findings suggest that SBRT represents an effective local treatment option for carefully selected patients but should be integrated with contemporary systemic therapies within a multidisciplinary treatment strategy. Prospective studies incorporating molecular biomarkers and modern systemic treatments are needed to better define the role of SBRT in this rare clinical setting.

## CRediT authorship contribution statement

**Burak Tilki:** Writing – review & editing, Supervision, Methodology, Conceptualization. **Ozan Cem Guler:** Writing – original draft, Visualization, Investigation, Formal analysis, Data curation. **Birhan Demirhan:** Writing – review & editing, Investigation, Data curation. **Pervin Hurmuz:** Writing – review & editing, Investigation, Data curation. **Ayşenur Elmalı:** Writing – review & editing, Investigation, Data curation. **Tolunay Kumru:** Writing – review & editing, Investigation, Data curation. **Gokhan Ozyigit:** Writing – review & editing, Supervision, Methodology. **Melek Yavuz:** Writing – review & editing, Investigation, Data curation. **Cem Onal:** Writing – review & editing, Writing – original draft, Supervision, Project administration, Methodology, Formal analysis, Conceptualization.

## Declaration of competing interest

The authors declare that they have no known competing financial interests or personal relationships that could have appeared to influence the work reported in this paper.
